# Foreign

**Published:** 1840-10-17

**Authors:** 


### FOREIGN.

*On the Epidemic Puerperal Fever of* 1838. By M. Voillemier.—Two distinct forms of puerperal fever were met with by M. Voillemier; the first of an inflammatory, the second of a typhoid type.

The inflammatory form made its attack by a slight rigor, soon succeeded by intense pain in the hypogastric region, or in the *iliac fossa*, in general limited to one spot. General reaction soon succeeded, and the pulse, which during the rigor was depressed, rose to one hundred and thirty beats in the minute or more. The skin became hot and covered with perspiration; the face became flushed; and the eyes lustrous. Frontal headach, often very intense, soon followed, and the breathing became hurried. This first period terminated by copious perspiration; occasionally, however, reaction did not commence till after the employment of copious depletion.

This form of puerperal fever in general yielded to the antiphlogistic regimen, though it occasionally terminated fatally; but it was by no means so intractable or fatal as the typhoid form.

The typhoid form of puerperal fever began with a long and severe rigor, which often came on a few hours after delivery. The pain in this form extended over the whole abdomen, and was often so intense that the weight of a cataplasm was insupportable. The abdomen became rapidly swollen; the pulse was feeble, undulating, very easily compressed, about one hundred and fifty in the minute, often so quick as to be countdd with difficulty. The respiration was hurried, and the anxiety extreme. There was intense frontal headach; the countenance was sunk; the face pale, and covered with a clammy sweat, which gave it the appearance of being varnished. Soon after this constant purging and vomiting wore out the patient. The matters vomited were of a green colour, and the feculent discharges were very fetid. Death usually occurred at the end of a few days, sometimes of a few hours. The patients were generally sensible to the last.

In the inflammatory form the bowels were constipated, whilst diarrhœa prevailed in the typhoid type. In neither of the forms did the suppression or continuance of the flow of the lochial discharge, or that of the milk, appear to have any marked influence on the disease.

The morbid appearances were as follow. In twenty-two out of twenty-four cases purulent matter was found infiltrated in the sub-peritoneal cellular tissue and in that of the pelvis. In two cases purulent inflammation of the lymphatic vessels, but of small extent, was met with. In three cases uterine phlebitis was noticed. The uterus itself was in most cases free from all marks of lesion. In a few cases small collections of purulent matter of the size of a pea were found scattered through its substance, but never an abscess of any size. These were in general nearer the external than the internal surface of that organ. Purulent infiltration was more frequently met with in the sub-peritoneal cellular tissue of the uterus, and that which occupies the pelvic cavity. This tissue, in fact, was in some cases so loaded with purulent matter, that when cut through it presented a greenish surface, and in many points abscesses of the size of a chestnut were met with. In six cases the peritoneal coat appeared healthy. Various morbid appearances were observed on this membrane; its vessels were found injected, presenting various arborescent forms; its surface was found covered with a serous fluid, more or less transparent, but more generally puriform; purulent matter and false membranes were also often observed over its surface. In one case there was perforation of the stomach. In three cases abscesses in the muscular parts of the arm or leg were met with; in one case purulent matter was found in the elbow and wrist joints; and in six cases purulent deposites were noticed in the cavity of the pleura.

M. Voillemier could not distinctly trace these diseases to the direct action of cold or any peculiar atmospherical state. He, however, regarded as predisposing causes, close apartments, want of ventilation, an impure atmosphere, and elevated temperature; also insufficient or bad food, privations of all kinds, and neglect of the state of the body during pregnancy. He thought that in a few cases he could trace the propagation of the disease to contagion. Those who were delivered by means of instruments appeared to be more particularly subject to the disease. Of fourteen instrumental cases, six were attacked with this puerperal fever, and four died.

M. Voillemier in conclusion remarks, that "puerperal fever may be considered as a disease essentially general, of which the anatomical character consists in the existence of purulent matter in some part or other of the body."

As to the treatment, he places the greatest confidence in the antiphlogistic regimen, viz., the local and general abstraction of blood; cataplasms to the abdomen; injections when the lochia are fetid; and purgatives, especially in the inflammatory form. He thinks he has seen two instances of persons owing their lives to mercurial friction.—*Edinburgh Medical and Surgical Journal, from Journal des Connaissances Medico-Chirurgicales*, December, 1839, January, 1840.

---

*Case of Salivation from the use of Hydriodate of Potash.* Ry Sir Francis W. Smith.—A young man had been treated in the Hospital about a year and a half before, for the venereal disease, but was dismissed without being cured. For the five weeks previous to his being seen by Sir F. Smith, he had been taking Plummer's pill. He was much reduced in flesh and strength, and had a bad cough; he suffered from pains in the shins and scapulæ; had sore throat, and an eruption of a copper-colour upon his arms and shoulders. He also complained of tenderness, and even pain in the frontal sinus and bones of the nose, from which dark-coloured matter and crusts, but without any peculiar fetor, were discharged. He was ordered sarsaparilla in powder, and ten grains of the hydriodate of potash, gradually increased to fifteen daily. By three weeks his cough nearly left him, and he had increased greatly in flesh and strength. He complained less of the pain or tenderness in the frontal sinuses, and the discharge from the nose had ceased. His appetite was improved and he slept well. But what was most remarkable, was that he was salivating freely, and his front teeth were all loose, as if he had been undergoing a course of mercury, with this difference, however, that there was no fetor of the breath.—*Ibid., from Dublin Journal of Medical Science*, July, 1840.

---

*Twelve Tæniæ expelled by a decoction of the Pomegranate Root Bark.* By Dr. Mongeal.—A lady, thirty-two years of age, and having all the appearance of health, had complained for seven or eight months of general uneasiness and feeling of weight in the epigastric region; at times also her abdomen became suddenly swollen. She had, in general, a disgust at food; occasionally, however, her appetite was inordinate; she had frequently desire to vomit, but never vomited. Her tongue was enlarged and covered with a white fur. She complained of an occasional pricking or tearing pain in the region of the stomach; and when she ran she felt a body which she compared to a bladder full of water moving up and down in the epigastric region. She had frequent attacks of diarrhœa. Twice she had had convulsive fits of an hour's duration during the night, attended with loss of consciousness, but had no recollection of them when she awoke from sleep next morning.

As from these symptoms the existence of intestinal worms was suspected, the stools were carefully examined for some days, when a few joints of a tænia were discovered. A strong decoction of the root-bark of the pomegrante was therefore administered, and about an hour afterwards a large knotted mass of tæniæ was suddenly evacuated. On unrolling this mass twelve separate worms were discovered, and their aggregate length amounted to one hundred and fifty-seven English feet.

The unpleasant symptoms immediately afterwards disappeared.—*Ibid.*, *from Archives Generales de Medicine*, July, 1840.

---

*Case of Ischuria Renalis of nine years standing, with Vicarious Vomiting of Urine.* By Dr. F. L. Kreysig.—A woman, twenty-five years of age, began to complain of abdominal pain, general spasm, difficulty in voiding urine and feces, and afterwards of orthopnœa and symptoms of thoracic and abdominal inflammation, which were treated antiphlogistically. The urine required to be drawn off by the catheter. She also suffered pain in the right knee, and generally kept that limb drawn up towards the abdomen. Two years afterwards, the legs began to swell, the respiration became more difficult, the urinary secretion ceased, and she vomited from time to time a fluid containing, according to chemical analysis, the principles of urine.

Dr. Kreysig now detected in the right iliac region, near the spine, a tumour extending towards the liver, and exquisitely painful to the touch. The patient had observed this tumour four years previously. The bowels were costive, and twice a-day urinous vomitings occurred, accompanied by fits of dyspnœa, so severe as twice to require the aid of venesection to relieve them. The bladder was empty. Tepid baths were now used, and mercurial and stimulant liniments applied to the region of the kidney. When the patient was in the bath her skin exhaled a very fetid odour.

The tumour continued to enlarge, and four months after it became harder and more painful, and pointed in the epigastric region. Three weeks afterwards, during a violent spasm, it burst, and discharged a large quantity of purulent matter, accompanied with a great increase of all her sufferings. Six weeks from this period the urinous vomiting ceased, and she began to pass urine by the natural passages. Shortly afterwards, during a spasmodic attack of pain, and an urgent desire to evacuate the bowels, she passed a mass as large as a goose's egg, resembling fat mixed with purulent matter, and intolerably fetid. From this period the abdominal pain and swelling disappeared, and menstruation, which had been stopped during the period of her illness, returned, and within nine months her health was completely re-established.—*Ibid.*, *from Hufeland's Journal*, July, 1839.

---

*On Sulphate of Copper as an Emetic.* By Dr. A. Toulmouche.—As much uncertainty seemed to prevail with regard to the sulphate of copper being a safe emetic, and also regarding its dose when administered with this intent, Dr. Toulmouche administered it in varied doses to 72 patients, and the following are the results he obtained.

The dose of 10 centigrammes (1.5 grs. English) was given to 12 women, and in nine of them produced vomiting. Five vomited once; one vomited twice; three vomited thrice; and two four times. It also produced purging in four of the cases.

In the dose of 20 centigrammes (3 grs.) it was administered to 36 women, and excited vomiting in 32; and the average number of times each vomited was thrice. It also acted on the bowels in 23 of the cases, causing from one to three alvine evacuations.

In the same dose it was administered to five men, and in all it produced violent vomiting, eight times in each. In four it acted also slightly on the bowels.

To eight women it was given in the dose of 30 centigrammes, (4.5 grs.) and it caused vomiting in all three times; it caused purging in six cases.

To eight women it was administered in the dose of 40 centigrammes (6 grs.) and excited vomiting in all to the number of from three to four times; and in four it produced alvine evacuations.

When administered in the same dose to two men it produced vomiting in one only, and one stool; on the other it had no obvious effect.

Lastly, it was given to one woman in the dose of 60 centigrammes (9 grs.) but no effect was produced by it. In nearly one-half of the above cases it caused griping.

M. Toulmouche from these cases concludes that it is a sure and safe emetic; and in no respect more dangerous than the preparations of antimony; but that its purgative effect cannot be depended on.—*Ibid.*, *from Gazette Medicale de Paris*, 23d May, 1840.

*On Sulphate of Zinc as an Emetic.* By Dr. A. Toulmouche.—Dr. Toulmouche shows some want of information as to what is known in this country with regard to the effects and doses of medicines. He never ventured to administer this medicine in doses above 75 centigrammes, or 11.5 grains English; it is, therefore, not wonderful that he found it to be a very inferior emetic; no fewer than 33 cases failing out of 83 to whom it was administered. The only wonder is that it succeeded so often; but the fact may be explained in this way, that the greater portion of the individuals to whom it was administered were labouring under "embarras gastrique," and such being the case, the nauseous metallic taste of the medicine would be of itself quite sufficient to excite an evacuation of the contents of the stomach. He expresses great astonishment at the doses which English practitioners are said sometimes to give, viz. 100 centigrammes to 2 grammes, (15 to 30 grains,) but explains it by supposing it could only have been in cases of poisoning by opium. In our own experience sulphate of zinc has been found to be a very safe and efficacious emetic in the dose of 20 to 25 grains, neither exciting nausea nor much retching; in fact, it is the most pleasant emetic with which we are acquainted. —*Ibid.*, *from Gazette Medicale de Paris*, 6th June, 1840.

*On the Hydrocyanoferrate of Quinine.*—Notwithstanding the valuable febrifuge virtues of the sulphate of quinine, it is well known that it occasionally fails. In such cases the hydrocyanate of quinine has been used with good effects. But as this is a salt subject to decomposition, Signor Bertozzi, of Cremóna, has proposed to substitue the hydrocyanoferrate of quinine, whose powers over the worst forms of intermittent fever have been completely established.

Dr. Zaccarelli has prescribed this new medicine in a great number of cases in place of sulphate of quinine. It is found to cut short tertian and quartan fevers; and, what is well worthy the attention of physicians, it has principally succeeded in cases where the sulphate of quinine has failed. Dr. Carioli has also confirmed the febrifuge properties of this preparation.

The following is given by Bertozzi as the most economical process for obtaining it. One part of sulphate of quinine is to be triturated in a glass mortar to an impalpable powder; a part and a half of the ferro-prussiate of potash, previously dissolved in six or seven parts of distilled water, is to be mixed by careful agitation, and the whole exposed in a flask to heat, stirring the mixture carefully until it arrives at the boiling point. In proportion as the liquid becomes transparent, there is precipitated to the bottom and sides of the flask a substance of a greenish-yellow colour, having an oily consistence. Having poured off the liquid portion, this substance is to be washed with distilled water, and then dissolved in very pure alcohol at 100° Fahr., and immediately filtered. On evaporating the alcohol, a mass, confusedly crystallized in needles, is left, the weight of which amounts to three-fourths of the sulphate of quinine used. This is the hydrocyanoferrate of quinine.

When in small fragments this substance is of a pea-green colour, and of an intensely bitter taste. It dissolves in cold, but better in hot alcohol, and is precipitated from its solution almost entirely by water. It is decomposed by sulphuric acid, and by the tincture, infusion, and decoction of cinchona. It has been given in doses of three grains and a half, repeated as occasion required.—*Ibid.*, *from Dublin Journal of Medical Science*, July, 1840.

*On Subcutaneous Wounds of the Joints.*—By Dr. Jules Guerin.—In a preceding memoir, M. Guerin sought to establish the fact, that wounds not involving the skin, and, therefore, kept from exposure to the air, were not followed by inflammatory symptoms, and were usually healed by the first intention. In this paper, M. Guerin endeavours to show from experiments on man and the lower animals, that subcutaneous wounds of the joints, (*i. e.* those not involving the skin,) as of the tendons, muscles, aponeuroses, cellular tissue, nerves, and minute vessels, may all, by means of particular precautions, be healed by the first intention.

His first experiments were made on animals. He opened successively on two dogs, by the subcutaneous method, the humero-cubital, radio-carpal, femoro-tibial, and tibio-tarsal articulations; and when no air was allowed to come in contact with the opened joint, they healed immediately without exhibiting any traces of inflammatory action. When these articulations were, however, left free in their motions, synovial tumours formed around the joint: but if they were kept in a state of repose, and permanent extension, the cure was completed without any accident whatever. When the wounds were made so as to allow of the contact of air, or its introduction into the joint, inflammation and suppuration were excited, proportioned in extent and intensity to the duration of the contact of the air.

His next experiments were on man. Acting on the results furnished by these experiments, and also on what is every day observed in man, where, in luxation of the shoulder or hip-joints, though considerable injury is inflicted on the capsular ligament and neighbouring parts, no inflammatory symptoms are set up, provided the skin be not torn, he ventured to operate on man. He has many times made a subcutaneous section of the ligaments, and a portion of the fibrous capsule of the knee, and of the foot, to remedy deformities of these joints; and in no instance have these operations been followed by inflammatory action.

The precautions which must be taken in order to insure the subcutaneous wounds of the joints from inflammatory accidents, are, to make the aperture in the skin very small, and as far as possible from the articulation; to make it while the limb is extended, and not when it is bent; and to keep the limb after the operation in the most perfect state of repose. These two last directions are the consequences of a theory which M. Guerin has endeavoured to establish, which is, that articulations are, during their movements, the seat of a partial temporary enlargement, which causes in them a tendency to produce a void, so that if the limb was flexed or extended after the operation, a suction takes place at the orifice of the wound, and air is drawn into the joint.

In the third part of his paper, M. Guerin shows the practical uses of his discovery, in the art of surgery. Serous, sanguineous, and purulent collections of matter in the articulations may, by the same means, be drawn off with safety. But it is chiefly to its advantages in aiding the reduction of old luxations, or removing deformities by incising the ligaments or tendons, that M. Guerin directs the attention. He has already by this means cured a congenital luxation of the clavicle which had resisted all known means. He made numerous incisions of the ligamentary apparatus around the head of the bone, and after two operations, so conducted, succeeded in bringing it into its proper place, and curing completely the deformity.—*Ibid.*, *from Seances de l'Academie des Sciences*, 4th May, 1840.

---

*Practical observations on peculiar affections of the throat, arising from abscess between the pharynx and spine, and occurring in children and adults; exemplified by cases.* By Christopher Fleming, M. D.—The several obstructions, mechanical or otherwise, which occur in the fauces, and impede the functions of respiration or deglutition, have particularly attracted the attention of the profession. They are frequently met with, and in the majority of instances they are referrible to causes sufficiently manifest. Occasionally, however, considerable difficulty attends their diagnosis, particularly in children, from the extreme obscurity and anomalous character of the symptoms. Such difficulty occurred in those attendant on inflammation at the back of the pharynx, terminating in abscess, illustrative of which I beg to subjoin the following cases.

Of a family of five boys the eldest, aged seven years, the youngest one year and eight months, three were attacked as follows, without any assignable cause.

The youngest, a healthy child, went to bed well; after about two hours awoke with vomiting, which attracted no particular attention; passed the night tranquilly; next morning appeared heavy, took his ordinary mid-day sleep' and was found, about two o'clock, P. M., in convulsions. Immediate assistance was procured, which, notwithstanding the most prompt and active treatment, proved unavailing. I saw him for the first time about two hours before death; he was then comatose, and almost pulseless; the left side was wholly paralytic; the right slightly convulsed. He survived the attack only twenty-two hours, dating from the supervention of the convulsions—thirty-nine from that of the vomiting.

On examination after death, considerable vascular turgescence was found within the skull, and throughout the substance of the brain. No other appreciable lesion was discernible.

This occurred on the last Friday in May, 1836.

On the following Sunday, the third boy, aged six years, was attacked. He was a remarkably delicate child, and much emaciated, being then only convalescent from remittent fever. He now vomited repeatedly, complained of violent pain in the head, and had other smart febrile symptoms. However, by mild depletory measures, he passed through an illness of three or four days' duration, without any remarkable occurrence.

On Monday, the fourth boy (the subject of this communication) sickened with precisely the same train of symptoms. His age was three years and a half, and in appearance he was healthy. The premonitory symptoms of his attack, at first mild, after about thirty-six hours assumed most intense severity, and without unnecessarily particularizing their progress, it may be stated, that the most aggravated form of high inflammatory fever set in, principally engaging the cerebral organs, and requiring the most energetic treatment to combat it. On about the fourth day, convalescence appeared established, and Dr. Crampton (whose valuable assistance I had throughout the progress of this case) discontinued his daily attendance.

From day to day a peculiar fixed position of the head, and stiffness in the neck, now attracted attention. The head was drawn back. The muscles, at first tense, became completely and permanently rigid, and the movements of the head painful, and remarkably limited. Soreness of the throat was complained of, and also great difficulty in swallowing, at times accompanied with violent spasmodic efforts. There was no cough, and the voice remained perfect. The articulation became remarkable—the words being as if drawled out with pain and difficulty, and at times perfectly unintelligible.

Repeated and careful examination of the fauces and neck could not detect any apparent local cause for those symptoms, which, with varied degrees of intensity, advanced, producing equally alarming constitutional disturbance and debility.

At first, disposed to attribute them to concurrent local causes, such as the quantity of mercury administered during the acute illness of the child, the cold from the renewed application of ice to the head, or some partial internal effusion, the result of the acute inflammatory attack, more serious mischief was now apprehended from their increasing severity and permanency. The treatment adopted was principally with the view of promoting the absorption of any fluid effused, and consisted chiefly in the exhibition of mild mercurial alteratives, and the application of counter-irritants to the region of the occiput.

On about the tenth day, the symptoms had reached their acmé; the child, emaciated and weakened, had no relish for food, and appeared to drink merely to allay thirst, the efforts at swallowing being convulsive and painful. He was now in a perfect state of somnolency, regardless of every thing about him, when accidentally, while setting beside his bed, I perceived that *position* most remarkably influenced the severity of the prominent symptoms. Stupor in the recumbent posture, almost amounting to perfect coma, in the sitting, or even semi-erect, resolved itself into a comparative sensibility. Respiration, slow, laboured, and stertorous, or rather roaring, (as described by the attendants on the child,) in the former position, became comparatively tranquil in the latter, and a pulse, in the one, ranging only a beat or so above forty, in the other assumed a more natural character. Again, fluids were more frequently darted convulsively forwards through the nostrils or mouth, than passed into the stomach, or were ejected, as in the act of vomiting, and the recurrence of the symptoms of cerebral compression took place on returning to the recumbent posture, which for the last three days had been almost the permanent one.

I now considered that this relation of symptoms might still be caused by mechanical obstruction in the pharynx, although repeated examinations on former occasions did not lead me to this conclusion. An additional obstacle presented itself in the fixed position of the jaws, so that it was only by considerable force I could so far separate them as to admit of even getting my little finger between them. On forcing it back, I accidentally, but distinctly, felt a tumefaction beyond the base of the tongue, giving, as well as a compressed finger could indicate it, a sense of yielding. To get a view of it was utterly impossible. The soft palate and uvula were easily discernible, but the depression of the tongue gave so much pain, and the separation of the jaws was so very limited, that further investigation was totally out of the question. Indeed, in addition, the evidence, even from touch, was necessarily momentary, from the severe paroxysms of dyspnœa attendant on the examination. Although I had never heard of, nor witnessed, a case of the kind before in children, it at once occurred to me that this might be an abscess at the back of the pharynx, mechanically producing the above symptoms, and having stated this as my opinion to the family, the assistance of Dr. Crampton and Mr. Cusack was immediately procured. After a patient, though extremely unsatisfactory examination, they coincided in opinion with me as to the presence of a tumor in the situation alluded to, and it was determined that I should perforate it with an explorator which I had provided for the purpose, with the view of ascertaining its actual nature,—a doubt existing on this head, not alone from the extreme firmness of the tumor communicating a very indistinct sense of flunctuation, but also on account of its probable anomalous nature, from the previous acute and present chronic cephalic symptoms. With every necessary precaution I accomplished this object, though with considerable difficulty, and, to my great gratification, witnessed the sudden gushing forth of a large quantity of healthy purulent matter. The whole features of the case were almost instantaneously altered. The somnolency was removed, deglutition was facilitated, and more cheering prospects manifested themselves.—Nourishment was freely given throughout the day, and quinine administered in small and repeated doses.

At my evening visit I perceived that the stertorous breathing had returned, and that the more prominent symptoms, which had ceased since the operation, were again in some degree present. I examined the throat, and fortunately found the separation of the jaws now accomplished with ease. The abscess was again filled, with the opening closed. I introduced a carefully protected sharp-pointed bistoury into the site of the opening, and freely enlarged it downwards. The relief was instantaneous. I now directed the trunk of the child to be elevated as much as possible, and the head depressed. The night was passed comparatively tranquil; the quantity of matter which escaped through the mouth was considerable, largely staining the pillow. The next day the boy was able to play with his brothers, and subsequently his improvement was progressive, though slow.

He is now a fine healthy boy. I do not particularize the treatment adopted during his convalescence; there was nothing peculiar in it, its principal object being to improve the general health. The next case which I shall select is that of a boy aged seven months, proving the remarkable fact of the occurrence of such an affection during the first period of childhood, as the former does during the second.

In April, 1838, I was sent for to see this child by the father, who stated that he had great apprehension his little boy was labouring under water on the brain; that many children of his immediate family had fallen victims to it, and that the symptoms under which this child laboured were exactly those by which the attacks of the former had been ushered in. On visiting the child I found every indication of gastro-enteric derangement, so common at this period of life, and very suspicious cerebral complication, rendered more so from the fact of hereditary predisposition. In addition, I found that some lymphatic glands, on the left side of the neck, near the angle of the jaw, were enlarged and painful, evidently depending on ulceration behind the corresponding ear. The mouth, fauces, and pharynx, were free from lesion, and one of the incisors on the lower jaw had just made its appearance.

The treatment was principally directed to the abdominal system, and to the relief of the glandular irritation noted. After a few days, improvement was so manifest, that I had omitted a visit on Friday.

On Saturday morning I received a hurried message to see the child, and found that the more alarming symptoms had all returned during the previous night, that the restlessness was incessant, that the vomiting was constant, that the flushing of the face was renewed, that the breathing was loud, laboured, and very irregular during the night, and that he constantly started from most disturbed sleep, which would only be tolerated in the nurse's arms; that every attempt at putting him in the cradle aggravated the pulmonary symptoms. In addition, I observed that the head of the child was rather drawn back, and that the chin projected somewhat unnaturally. He immediately screamed when the jaws were attempted to be separated, and in the region of the neck there was the greatest tenderness, particularly over the glands above alluded to. The integuments were free from discoloration, yet still the tumefaction was decidedly increased, and the slightest motion of the head appeared to give great pain.

At the moment, I was disposed to attribute the recurrence of those symptoms to a smart attack of inflammation in these glands, and was led to hope that the combating it would relieve them. The treatment was accordingly directed with that object in view. Leeches were applied; fomentations and poultice used, and a smart mercurial purgative administered.

*Sunday.*—Night spent wretchedly; no alleviation of symptoms, with the exception of those connected with the inflamed glands; they are better: the other symptoms are, if possible, more aggravated. In addition to those enumerated in the report of yesterday, there is now a gurgling noise in the fauces, as if from accumulated mucus, and throughout the lungs there is evidence of considerable effusion into the larger bronchial tubes; there are repeated and apparently painful and difficult efforts at swallowing, accompanied with frightful paroxysms of dyspnœa occurring at irregular intervals, during which the countenance becomes suffused, purple, and almost convulsed; and it is remarked that those immediately supervene on attempting to place the child in the cradle; there is incapability of sucking, though great desire for the breast, the nipple of which is seized with avidity, and equally rapidly ejected with a sudden and spasmodic regurgitation of the milk. Any fluid placed in the mouth either remains for a short time, and then gradually dribbles out, or otherwise produces a paroxysm accompanied with similar phenomena. At the moment of my visit, the repeated exertions of the child at the attempt of swallowing, the severe dyspnœa, and the great accumulation of mucus in the fauces, with the very restless state of the child, led me to apprehend the supervention of a fit of convulsions. I thought I recognized some of the features of the above case, when, from some unintentional act in my examination, a most severe paroxysm supervened. The child appeared suffocating. I rapidly passed my finger into the fauces, and feeling a fulness, I made pressure against it, which was increased by a convulsive effort of the child. A sudden discharge of purulent matter got exit through the nostrils, and temporary relief was obtained, until I procured the additional assistance of Sir Henry Marsh and Mr. Cusack.

Perhaps about an hour or so had elapsed from the above occurrence when we met in consultation. At this time the breathing, though principally nasal, was more tranquil; and a small quantity of fluid had been swallowed, but with much difficulty. The appearance of the child could not but make an impression upon those who saw him. The nostrils were filled with matter which trickled down the lip; any attempt at placing him in a recumbent posture was instantly followed by frightful dyspnœa, rendered still more serious from the great accumulation of mucus in the fauces. I directed attention to the throat; but notwithstanding every effort, no accurate view could be had of the back of the pharynx. The narrow space behind the root of the tongue was filled with pus and bubbles of frothy tenacious saliva, to clear which away various repeated unsuccessful attempts were made. Here the freedom of separation of the jaws allowed of free, though rapid examination of the fauces, but the back of the pharynx could not be seen. I, however, felt a distinct tumefaction, and failing to puncture it with the grooved curette, as in the former case, I was obliged to rest satisfied with what had been done, arranging to watch the progress of the symptoms, and to support the child by every possible means, by introducing fluids through a tube passed through the nares, and by broth enemata; to be prepared, if necessary, to open the trachea should any fresh symptoms of suffocation supervene; and in addition, to keep constantly cleared away the accumulating phlegm at the back of the throat.

By visiting at short intervals, and carefully enforcing the above injunctions, the strength was supported, and the symptoms to a certain extent stayed. Next day they were stationary, though it was quite evident that considerable obstruction yet existed in the throat; however, the strength was improved, and the countenance of the child decidedly better. Another day passed without any material change, when the discharge from the nostrils ceased, and evidently, any opening made, or rather the ruptured portion of the sac, had closed. Difficult respiration in any but the erect posture, or on an inclined plane with the head considerably depressed, recurred. Perfect inability of sucking and swallowing again set in, and suffocation appeared impending, when Mr. Cusack saw the child, and was still more satisfied of the presence of a tumour at the back of the pharynx. It was so tense and so unyielding, that did not the history of the case justify the presumption that matter was present, the absence of any sense of fluctuation would have caused extreme doubt; another difficulty presented itself in its being below the level of the tongue. The very limited space to operate in, together with the risk of wounding the neighbouring vessels, on account of the disposition of the swelling rather from the median line towards the left side, suggested the propriety of selecting some instrument the action of which could be accurately gauged. That which I had used in the former case was objectionable, not alone from the want of sufficient command of it from its conformation, but also from its shape. It was agreed that delay might be safely hazarded until next day, leaving word, however, that should any urgent symptoms set in, I should be informed.

Next day, I found that throughout the night great apprehensions were entertained lest suffocation should have taken place. All other bad symptoms remained, if not aggravated, at least stationary; and having arranged in the interim with Mr. Cusack, an instrument was contrived which succeeded most admirably. It consisted of a trochar about four inches long, one extremity of the canula being slightly curved, the other with a ring on its upper surface to receive the fore-finger; into this canula was passed a jointed stilette, with, at its opposite extremity, a ring for the thumb, and a moveable screw to graduate the projection of its point. Mr. Cusack having firmly supported the head of the child, I passed the fore-finger of the left hand towards the back of the pharynx, there resting the point of it, and guiding the armed trochar with the concealed stilette along it, accurately fixed it on the tumour, pressed forwards the stilette to its limited mark, and withdrawing it by an opposite manœuvre, was gratified to see *a quantity of healthy purulent matter darted forwards on the child's clothes.*

The relief was immediate; the hæmorrhage trifling; and the result permanently successful. In this case it was unnecessary to renew the opening; the discharge, at first temporarily ceasing, returned, and the cure was rapid.

The boy is now a fine, healthy boy. The constitutional treatment was similar to that adopted in the last case.

Such is the history of two extreme cases of *acute* abscesses at the back of the pharynx, occurring in children, selected from others of the same nature, which I have witnessed within the last three years, and necessarily with opportunities comparatively limited. I have brought them forward as remarkably illustrative of the symptoms attendant on their progress; as novel at that period of life, in the records of medicine, so far as I have been enabled to learn, from the investigations I have made; and as corroborated by the testimony of others.

I cannot instance the history of any similar acute case occurring in the adult, which came immediately under my observation, although I have watched for such with much anxiety for no very short period. I have attended many severe cases of tonsilitis, which have terminated in suppuration, some of which I have opened between the pillars of the fauces, and some on the anterior part of the vellum. I have met with abscesses of the vellum itself, and of the uvula, and I have met with one or two of that description, so accurately and so beautifully described by Petit*, which form *behind* the tonsil, and I believe always implicate more or less the auditory apparatus, but I have never been able to det[illegible]t an abscess situated distinctly at the back of the pharynx, or perhaps, I should rather say that the symptoms attendant on such did not attract my attention. That such collections take place cannot, however, be questioned. The experience of our surgeons in extensive practice will bear testimony to the fact of their occasional, though extremely rare occurrence, and will, I am sure, confirm the statement, that their attendant symptoms are so equivocal and anomalous, that if discovered, they have been so by the merest accident. The first systematic author I find particularly alluding to their presence, is Sir Astley Cooper. In his lecture on Abscesses, he thus expresses himself: "Abscesses are also dangerous, from their being situated in vitally important parts, such as the brain, heart, or lungs; or when they are not seated in parts of vital importance, from their pressure on essential organs.

"Cases.—A woman was admitted into Guy's Hospital for a complaint in the throat, occasioned by *swallowing a pointed bone*. All she complained of at first was a soreness in the throat; but she was shortly after seized with difficulty of breathing, which increased greatly, and she died.

On examination after death, I found, upon making an incision into the pharynx, that *between it and the forepart of the vertebræ*, a large

*Traité des Maladies Chirurgicales, et des Operations qui leur conviennent. Ouvrage Posthume de M. J. L. Petit. Paris Edition, 1774. Chapitre iv. des Tumeurs, p. 139.

abscess had formed, which, by pressing the pharynx forward on the epiglottis and glottis, occasioned difficulty of breathing, and in the end destruction of life. Shortly after this, Dr. Babington came to this hospital with a friend of his, who was labouring under great difficulty of breathing. He requested me to examine his throat. Having put my finger on the back of the pharynx, and felt fluctuation there, I told him that this was a case of which I had seen *an instance*, where the patient had died from a collection of matter formed in the same situation. I immediately procured a seton needle, and including it in a canula, like a trochar, I put it down into the pharynx, let out a considerable quantity of matter, and the patient was relieved. Here was a case which, but for this operation, would probably have terminated fatally, by the pressure of the matter on vitally important parts."

In the "Dictionnaire de Medècine et Chirurgie Pratiques," under the article "Pharyngotome et Pharyngotomie," another case will be found, in which the presence of an abscess at the back of the pharynx was detected, and its puncture followed by successful results. But in each and all of those recorded cases, it is a remarkable fact, that the abscess was actually formed, before a suspicion of its existence was entertained, so extremely equivocal were its premonitory symptoms, even in the case where the exciting cause naturally led to the examination of its immediate seat. Hence, it appears to me, that the subject is one of extreme importance, and fully deserving of separate investigation.

(*To be concluded in our next.*)

---

*Cases of Perforation connecting the Œsophagus with the Air-Tubes of the Lungs.*—Dr. Osborne lately presented to the Dublin Association of Physicians, two preparations of perforations connecting the œsophagus with the air-tubes of the lungs.

The first (of which a notice has already appeared in the *Dublin Medical Press*) was taken from a young man, who enjoyed good health till about four months previous to his death. At that time he was first observed to cough always after swallowing liquids. This increased so much that he was at length obliged to abstain entirely from drinking. On the morning of his death he was eating a breakfast of beefsteak, when he was seized with a fit of suffocation, and was brought to the hospital gasping for breath. A probang was passed, with a view to remove the obstruction, but no improvement was obtained, and in a few minutes he died.

On examination after death nothing was found in the larynx or œsophagus to account for this sudden catastrophe, and the parts were on the point of being closed, when one of the assistants happening to put his finger down the œsophagus, felt something rough at the side of it, adjacent to the bifurcation of the trachea. This produced a further inquiry, and it was ascertained that a perforation had taken place exactly in the raphè above the bifurcation, that a portion of a piece of gristly beef had passed through it, which unfortunately for the patient had been divided into two portions, one of which had stopped up each of the bronchi, while it had been retained in its situation by a large portion connected with it, which remained in the œsophagus. The orifice through which it had passed was a longitudinal slit, which, when expanded, would form an opening equal to a fourpenny piece. There were no vestiges whatever of ulceration, or of any diseased process around the orifice; and Dr. Osborne stated his opinion to be, that this was a case of congenital defect resembling cleft palate; that owing to some accidental circumstance it had become enlarged four months ago; when swallowing fluids was followed by a cough, and the irritation attendant on their escape into the bronchial tubes; that the immediate cause of death was an irritation producing a necessity for coughing, unfortunately at the moment when the piece of beef had arrived opposite the orifice, when the unfortunate patient being obliged to make a sudden and forced inspiration preparatory to the cough, the beef was sucked into the trachea, and each lobe of it also sucked into a bronchial tube, which were thus as it were *corked up*. Perhaps there is not on record a more remarkable instance of loss of life from so singular a combination of circumstances: 1st, A perforation of the œsophagus penetrating the trachea just above the bifurcation; 2dly, The patient making a forced inspiration just at the moment when a piece of meat was in the act of passing the perforation; and 3rdly, The meat happening to be divided into three lobes, one stopping up each bronchial tube, while the third remained in the œsophagus, and thus maintained it in its position.

The second case was that of an individual who appeared to have possessed intellectual energies far above his station in life. He was a working shoemaker, and burthened with a family, but found time to combine the labours of authorship in various departments, with those of a political orator, and methodist preacher. About eleven months previous to his death he first experienced a difficulty in swallowing, which gradually became painful. When my patient was admitted into the hospital, he complained of lancinating pains in the œsophagus, even when not engaged in swallowing, which usually belong to the progress of scirrhous and cancerous disease in that part. The passage of the œsophagus tube, however, in the hands of one of the ablest surgeons of this city, failed to detect any, even the slightest stricture. Under treatment the state of his stomach was much improved, but no improvement of deglutition obtained, and he went to the country. His condition did not become sensibly worse till about seven weeks before his death: then he was tormented with cough on every occasion of swallowing, or whenever he lay on the left side, and continued in this state of suffocation till his death.

An opportunity of examining the body having been offered by the family, a very remarkable correspondence between the symptoms and the disease was brought to light. The greater part of the œsophagus was so much diseased that in the preparation it can scarcely be recognised. The ulcerations in several places formed large excavations in the mass of adjacent scirrhous structure. In the portion adjacent to the bronchial tube, a peculiarly large excavation has extended, engaging in it several of the bronchial glands, and here is an oval perforation with thin jagged edges, forming a direct communication between the œsophagus and air-tube. The size of this being nearly one inch in length and half an inch in breadth, we can readily understand: 1st, How swallowing any thing solid or fluid produced coughing; and 2dly, How some degree of ease was obtained by lying on the right side. It is also to be observed, that although both lungs were healthy, yet that at the apex of the left lung a collection of miliary tubercles were in progress of formation; this being a result of the peculiar irritation to which that lung was exposed, analagous to the case described by Dupuytren, in which there were no tubercles in the body, except round a pin adherent in the lung.

But those cases illustrate a lesion which has not been described in any work that has come into my hands, and which must be of great rarity, since no preparation of it is in the museum of the College of Surgeons, or in that of any other collection respecting which I have made inquiries. Perhaps, however, it has not been sufficiently sought for, and the communication of those cases to the Association may be useful, if it excites attention to the subject. As perforations between adjacent mucous membranes in other parts are not unfrequent—witness those between the vagina and rectum, the colon and stomach—this occurrence between tubes so closely adherent as the œsophagus, and air-tubes may be *à priori* expected to take place, and the above cases shewing that it actually does take place, it is to be inferred that the absence of observations of a similar kind has arisen from its having escaped observation, in consequence of pathologists having not sufficiently searched for it.—*Lon. Med. Gaz.*

---

*British Association, Sept. 22d.*—Dr. Charles W. Bell read a paper on Bronten d'Altepe, and Baghdad, in the East.

Dr. J. R. Cormack read a communication on air in the veins. He stated that his object in bringing the subject at all before the Section was, humbly to state what appeared to him a sufficient objection to the theory lately published by Sir C. Bell. He would not recapitulate details which he had already submitted to the profession at some length. Sir C. Bell believes that death is produced by the air acting detrimentally on the medulla oblongata,—that is, on the respiratory column of it. Dr. C. had slowly injected large quantities of air into the veins of animals, without causing death; and, indeed, unless much air *was quickly thrown in*, the animal did not die. Dr. C. farther stated, that in every case in which the experiment proved fatal, the right side of the heart was found enormously distended, and unable to contract, and he preferred considering this obvious and constantly to be observed lesion as the cause of death, rather than any thing founded on hypothesis, however ingeniously that hypothesis might be defended.

Dr. J. Reid believed that the views of Dr. Cormack were correct, and had seen many of the experiments referred to.

Dr. Pagan, of Glasgow, wished to know if Dr. C. thought that, in cases of traumatic gangrene, the air evolved might not prove fatal in the manner alluded to?

Dr. Cormack.—In some cases it may. But it would require a large quantity of air, and that suddenly evolved, to cause death in the manner described. I have discussed this question in the last chapter of my thesis. Dr. Cormack then read some medical notes regarding Tangier, in Barbary, which he had lately visited.

Dr. John Reid read a valuable paper on the Anatomy of the Medulla Oblongata. The object of this communication was to point out the relative position of the motor and sensitive columns of the spinal cord, as they pass through the medulla oblongata and pons varolii, and describe the attachment of the different motor and sensiferous nerves to these columns. Dr. Reid exhibited dissections, from which it appeared that the decussation of the pyramidal bodies is formed by the greater part, and in some cases by the whole of the fibres constituting these two eminences decussating with each other, and then passing into the posterior part of the middle column. None of these decussating fibres run into the anterior column of the opposite side, and there is no other decussation in the medulla oblongata besides this. On tracing the column which is connected with the olivary body, and which may be called the olivary column, we find that it passes downwards, approaches closely to the anterior medium fissure, immediately below the decussation of the pyramidal columns, and affords attachment to many of the roots of the motor nerves. On tracing this olivary column upwards, it is found to expand over the olivary body, affording origin to the hypoglossal and abducens along its anterior margin, and to the portio dura along its posterior margin. Part of this olivary column passes upwards to the corpora quadrigemina, affording origin to the smaller root of the fifth, and to the trochleator nerve. Dr. Reid also pointed out how the spinal accessory and part of the filaments of the par vagum may be connected with the motor column.—*Ibid.*

